# Maternal Diet, Behaviour and Offspring Skeletal Health

**DOI:** 10.3390/ijerph7041760

**Published:** 2010-04-16

**Authors:** Laura R. Goodfellow, Susannah Earl, Cyrus Cooper, Nicholas C. Harvey

**Affiliations:** Medical Research Council Epidemiology Resource Centre, University of Southampton School of Medicine, Southampton General Hospital, Southampton SO16 6YD, UK; E-Mails: lrg@mrc.soton.ac.uk (L.R.G.); se@mrc.soton.ac.uk (S.E.); cc@mrc.soton.ac.uk (C.C.)

**Keywords:** osteoporosis, epigenetic, early life origins, fracture, bone mass, vitamin D, neonate, fetus

## Abstract

Osteoporotic fracture has a major impact upon health, both in terms of acute and long term disability and economic cost. Peak bone mass, achieved in early adulthood, is a major determinant of osteoporosis risk in later life. Poor early growth predicts reduced bone mass, and so risk of fracture in later life. Maternal lifestyle, body build and 25(OH) vitamin D status predict offspring bone mass. Recent work has suggested epigenetic mechanisms as key to these observations. This review will explore the role of the early environment in determining later osteoporotic fracture risk.

## 1. The Burden of Osteoporotic Fracture

Osteoporosis is a skeletal disorder characterized by low bone mass and microarchitectural deterioration of bone tissue, with a consequent increase in bone fragility [1]. It is the commonest bone disorder in Western populations, and an important public health issue because of the potentially devastating consequences of fragility fracture [2]. Hip fractures are the most important consequence of osteoporosis; they require hospital admission and are associated with an excess mortality of 10–20% in the first year after the fracture [3]. In white women the one in six lifetime risk of hip fracture is greater than the one in nine risk of developing breast cancer [4]. The annual cost of all osteoporotic fractures to the United States has been estimated as $20 billion and to the European Union as $30 billion [5].

Osteoporotic fracture is a result of a combination of an injury generating a given force, and bone strength weak enough to fracture at that force. An established measure of bone strength is bone mass, which is a composite of bone size and bone volumetric mineral density [6]. The DXA derived measurements which contribute to bone mass are thus bone mineral content (BMC, g), bone area (BA, cm^2^), bone mineral density (BMD, g/cm^2^). True volumetric BMD (vBMD, g/cm^3^) cannot be directly measured by DXA, although estimates can be obtained by adjusting for body size.

Bone mass appears to track through childhood and adolescence to reach a peak in early adulthood, more through an increase of bone size than true volumetric density [7]. It then declines in older age, through loss of bone tissue (thinning of trabeculae and cortex), with an accelerated rate of decline at the menopause. Prospective studies in adults indicate that the risk of osteoporotic fracture increases continuously as BMD declines, with a 1.5–3 fold increase in the risk of osteoporotic fracture for each standard deviation fall in BMD [8]. The ability to predict fracture risk from BMD is at least as good, if not better, than the ability to predict heart disease from blood cholesterol levels, and stroke from blood pressure [9].

Preventative strategies against osteoporosis can be aimed at either optimizing the peak bone mass obtained, or reducing the rate of bone loss. Optimization of peak bone mass may be more amenable to public health strategies and has been shown in mathematical models to be a powerful predictor of age of onset of osteoporosis [6,10]. Although this crucial characteristic is partly inherited, the currently identified genetic markers only explain a small amount of the variation in individual peak bone mass and fracture risk [11]. Evidence is accruing that environmental factors may act early in development (in utero and early postnatal life), interacting with the genome to produce a persisting influence on postnatal skeletal development.

## 2. Skeletal Development

In order to understand the impact of environmental factors on osteoporosis risk it is necessary to understand fetal bone development. The bones of principal interest in terms of fragility fractures in later life are the long bones, which are formed by endochondral ossification. This process requires a pre-existing cartilaginous model, which starts to develop at 5 weeks gestation with the migration and condensation of mesenchymal cells in areas destined to form the bones [12]. These pre-cartilagenous anlagen reflect the size, shape, position and number of skeletal elements that will be present in the mature skeleton [13]. The mesenchymal cells then differentiate into chondrocytes, which form the cartilage. As the chondrocytes proliferate they produce an extracellular matrix, which facilitates angiogenesis and invasion of osteoblasts from the perichondrium. The proliferation of the chondrocytes has been shown to be influenced by parathyroid hormone related peptide (PTHrp) [14], cytokines of the IGF axis [15], activated vitamin D [16] and triodothryronine [17]. Once the osteoblasts have invaded the cartilage they lay down matrix, which is mineralized to form the bones.

The long bones then undergo a period of rapid cell division in the second trimester [6]; this is a “critical period” for long bone growth. “Critical periods” are specific periods of rapid cell division, which occur at different times during gestation in different tissues [18], for example the kidney has a critical period in the weeks immediately before birth. Tissues that are in critical periods are thought to be particularly susceptible to an adverse environment, such as a reduction in nutrients. Rickets has served as a longstanding example of an adverse early life environment, in the form of vitamin D deficiency, disrupting the process of bone development and untreated can lead to persistent changes in the child’s bone structure. It is now being realized that early adverse conditions have the potential to form “memory” which influences the risk of disease in adulthood too [19].

We will discuss the evidence for the impact of maternal behavior upon bone development, using six groups of studies: (1) retrospective cohort studies using data on the risk or prevalence of fracture in adults with comparison to their birth and/or early life records (as surrogate markers of early life nutrition and stresses); (2) mother-offspring cohorts in which the nutrition, body build and lifestyles of women before and during pregnancy were explored in relation to bone mass in their offspring; (3) detailed physiological studies that have explored candidate endocrine systems and age-related bone loss; (4) evidence of phenotypic responses to changes in environment from the natural world; (5) animal studies showing epigenetic modifications in response to controlled changes in environment, and finally emerging work concerning epigenetic changes associated with pathways that influence skeletal health in humans. Whilst discussing these groups of studies we will explore theories about the basis of such interactions.

## 3. Maternal Behavior and Bone Development

### 3.1. Retrospective Cohort Studies

The first evidence of a link between early life and osteoporosis risk came from an epidemiological study of 21-year-old women born in Bath from 1968 to 1969 [20]. Independent of adult weight and body mass index there was a statistically significant relationship between the girl’s weight at 1 year old and their adult BMC at the lumbar spine and femoral neck, important sites of osteoporotic fracture.

In the Hertfordshire cohort study the relationship between weight at 1 year and adult BMC remained when the subjects reached the age of 60–75 years, and was present in both sexes [21]. Using health visitor records the authors found a positive association between weight at 1 year and adult bone area at the spine and hip (*p* < 0.005). The relationships remained after adjustment for known genetic markers of osteoporosis risk, such as polymorphisms in the gene for the vitamin D receptor (VDR), and after adjustment for lifestyle characteristics in adulthood that might have influenced bone mass (physical activity, dietary calcium intake, cigarette smoking, and alcohol consumption). These findings corroborate observations from studies performed in United States, Australia, Sweden and the Netherlands [22].

Further analysis of the DXA scan images of the participants from the Hertfordshire cohort study showed a positive relationship between weight at 1 year and the inter-trochanteric width of the femur, but not femoral neck length [23]. The association remained after adjustment for adult body weight and was independent of proximal femoral BMC. This suggests that poor growth in utero and during the first year of life is associated with disproportion of the proximal femur in later life leading to a narrower neck but preserved axis length. This would correspond to a reduction in the mechanical strength of the region, over and above that attributable to BMC alone.

Twin studies provide an ideal opportunity for looking at the effect of the environment upon development whilst controlling for genetic variation [24]. In a study of 445 monozygotic (MZ) and 966 dizygotic (DZ) twins from the UK, at a mean age of 47 years, birth weight was found to positively predict BMC and BMD [25]. The MZ twins, despite being genetically identical, had greater intra-pair variability in birth weight than DZ twins. This highlights the crucial role of the intrauterine environment in development, as two-thirds of the MZ twins will have shared a placenta, whereas all the DZ twins will have had a placenta each. A majority of the MZ twins therefore had to compete for placental resources, with a subsequent compromise in their genetic growth potential. Furthermore, in the MZ twins compared to the DZ twins there was a stronger intra-pair association between birth weight and bone density. This is despite the MZ twins having the same genetic growth potential, and the DZ twins having differing genetic make-ups, which would act to widen the variability in their growth. This suggests that the early adverse environment of the smaller MZ twin, when competing for the resources of one placenta, strongly influenced the fetus’s future adult skeletal status.

It has now been shown that growth in utero and early life is associated with the actual risk of fracture, using data from the Helsinki Cohort Study [26]. Birth and childhood growth data from 7,000 men and women born in Helsinki University Central Hospital during 1924–1933 were linked to hospital discharge records for hip fracture (n = 112). After adjustment for age and gender three independent determinants of hip fracture risk were identified: tall maternal stature (p < 0.001), shortness at birth (p = 0.03) and low rate of childhood growth (height p = 0.006, weight = 0.01). Additionally, people who had hip fractures were more likely to be shorter at birth but of average height by age 7 years. This suggested that hip fracture risk may be particularly elevated among children in whom the growth of the skeletal envelope had been forced ahead of its capacity to mineralize. This could indicate a disadvantage of a mismatch between intrauterine and early life environments, with influences from both areas upon the eventual phenotype.

### 3.2. Mother-Offspring Studies and Developmental Origins of Health and Disease

There is a strong biological basis for a model of disease pathogenesis in which a single genotype can give rise to several different phenotypes, allowing the organism to adapt future generations to prevailing environmental conditions. This phenomenon is termed developmental plasticity [27]. Experimentalists have repeatedly demonstrated that alterations to the diet of pregnant animals can produce lasting changes in the offspring’s physiology and metabolism [28]. In humans the importance of the intrauterine environment was initially demonstrated by showing the increased risk of hypertension, coronary heart disease and diabetes with being at the lower end of normal compared to the upper end of normal birth weight [29–31]. The cohort studies above suggest a similar effect for the risk of osteoporosis.

The “thrifty phenotype” model suggests that the fetus becomes growth retarded in response to adverse environmental conditions in utero, and the associated adaptations induce a phenotype better suited to a deprived post natal environment [32]. This could be understood as a focus of resources on surviving early life, with a consequent diversion away from the functions of repair and protect. A striking example of the thrifty phenotype preparing undernourished fetuses for a harsh environment came from an Ethiopian famine exposed population, in which control children had a 9 times lower incidence of rickets than children with high birth weights [33]. This suggested that infants who had a harsh intrauterine environment were better able to cope with postnatal nutritional deprivation. The later life cost of neonatal adversity inducing a “thrifty” interaction with the environment has been investigated in a cohort born during the Dutch famine winter of 1944–45. As the cohort has advanced into middle age they have been shown to have higher rates a diverse range of diseases including coronary heart disease, diabetes, breast cancer and schizophrenia [34,35].

However, the thrifty phenotype model does not easily account for the graded effect on the risk of diseases, such as osteoporosis, seen across the range of normal birth weight, and in non-extreme conditions. The ‘developmental origins’ hypothesis extends the “thrifty phenotype hypothesis” to propose that an altered long-term risk of disease is the result of adaptive responses that the fetus or infant makes to cues from the mother about her health or physical status [36]. In this way small size at birth is not seen as causing a greater risk of osteoporosis, but rather both are outcomes of an adaptive response to a more difficult intrauterine environment [37].

The developmental origins hypothesis describes predictive adaptive responses being made at the phase of developmental plasticity (such as during intrauterine life, possibly during the “critical period”), to optimize the phenotype for the probable environment of the mature organism [27]. Mother-offspring cohorts provide an ideal opportunity to assess such interactions.

The first mother-offspring cohorts measured parents’ anthropometric and lifestyle characteristics and related this to their child’s neonatal bone mass [38]. After adjustment for gestational age and gender, neonatal bone mass was strongly positively associated with birth weight, birth length, and placental weight. Maternal triceps skinfold thickness at 28 weeks positively correlated with neonatal BMD, and mothers who smoked in pregnancy had, on average, babies with an 11% lower whole body BMC than mothers who did not smoke. Interestingly smoking at the time of the last menstrual period was not associated with neonatal BMC or BMD, suggesting a fundamental importance of the immediate intrauterine environment.

The Southampton Women’s Survey provided the opportunity to further investigate these interactions in a larger sample of 841 infants [39]. This confirmed that independent predictors of greater neonatal whole body bone area and BMC included greater maternal birthweight, height, parity, fat stores (triceps skinfold thickness), and lower physical activity in late pregnancy. Maternal smoking was again statistically significantly (and independently) associated with lower neonatal bone mass. These relationships were observed in both male and female neonates.

### 3.3. Physiological Studies

#### Nutrition

The mother-offspring cohorts also provide a platform to investigate the mechanism of interactions between maternal behavior and later bone health. Calcium and vitamin D, being key nutrients in the process of bone formation, are prime candidates for investigation. In the study of children involved in the nutrition in pregnancy study, bone mass in childhood was positively associated with corrected calcium level in the umbilical cord of the child at birth (r = 0.19, p = 0.02) [40]. In this study 31% of the mothers had insufficient and 18% had deficient circulating concentrations of 25(OH) vitamin D during late pregnancy (11–20 and <11 μg/L respectively). Lower concentrations of serum 25(OH) vitamin D in mothers during late pregnancy were also associated with reduced whole-body and lumbar spine BMC in children at age 9 years [40]. Maternal vitamin D status was statistically significantly associated with childhood bone area, and areal BMD. Estimated exposure to ultraviolet B radiation during late pregnancy (p < 0.0001) and the maternal use of vitamin D supplements (p = 0.01) both predicted maternal 25(OH) vitamin D concentration, and childhood bone mass (p = 0.03). Additional evidence supporting a role for maternal vitamin D status was obtained in the Southampton Women’s Survey, when maternal vitamin D concentrations again correlated with neonatal bone mass [41]. In developing populations, often the nutrient in short supply is calcium rather than vitamin D: Children of mothers, in a mother-offspring cohort study in Pune, India [42], who had a higher frequency of intake of calcium-rich foods during pregnancy had higher total and lumbar spine BMC and areal BMD, independent of parental size and bone composition. Circulating maternal 25(OH) vitamin D concentrations in this cohort were relatively high, and were not associated with childhood skeletal measures.

Studies of maternal nutrition have also been able to look beyond maternal calcium homeostasis to the broader relationship between maternal dietary pattern and childhood bone mass. In a Southampton cohort, principal component analysis was used to identify a pattern of dietary intake characterized by high intakes of fruit, vegetables, wholemeal bread, rice and pasta, and low intakes of confectionary, added sugar, white bread and crisps [43,44]. As this matched national guidance on health eating it was termed the “prudent diet”. Greater compliance with this “healthy” dietary pattern during pregnancy was associated with increased offspring whole body and lumbar spine BMD at age 9 years (p < 0.001), independent of maternal body build, smoking and physical activity [45].

It therefore appears that maternal diet during pregnancy is likely to have an effect on her offspring’s bone development. The question then arises as to whether this is a direct effect of the nutrients available for bone development, or mediated via an alternative pathway, and what other factors influence such pathways.

#### Endocrine Signaling

As described earlier the Growth Hormone–Insulin like Growth Factor 1 (GH-IGF-1) pathway is known to be involved in bone development. Cortisol, an endogenous glucocorticoid, influences the GH-IGF-1 pathway, as well as itself being skeletally active [45]. Physiological studies of hypopituitary patients indicate that low levels of growth hormone increase the risk of osteoporotic fracture [46], and exogenous glucocorticoids are well known to increase the risk of osteoporosis.

Developing chondrocytes have been found to express IGF-1 mRNA, and IGF-1 has been shown to stimulate their proliferation [47]. The placenta acts as a barrier between the maternal and fetal IGF systems, with fetal IGF predominantly produced in the fetal liver. Javaid *et al.* [48] found that umbilical cord serum IGF-1 concentration positively correlated with neonatal whole body BMC, BMD, lean mass and fat mass. Cord serum IGF-1 levels partly explained the effect of maternal smoking upon offspring skeletal development, but did not account for the other previously demonstrated parental characteristics, such as height, weight and fat mass. This suggested that both IGF-1 dependent and independent factors influence bone parameters of the neonate.

In adults profiles of circulating GH and cortisol were compared to bone density and birth records of patients in the Hertfordshire cohort study [49–51]. It was found that weight at 1 year positively correlated with median GH concentration and negatively correlated with cortisol concentration at age 61–72 years, suggesting a “memory” of a difficult intrauterine or early life environment. Furthermore the profiles of these two hormones were found to be determinants of prospectively determined bone loss rate, suggesting a physiological outcome of this “memory”. This suggests that the GH-IGF axis is instrumental in both the formation and maintenance of skeletal health, additionally the ability of this system to maintain skeletal health may be influenced by the events of intrauterine life.

#### Genetic variation

In order to dissect the relationship between fixed genetic variations in key genes, growth in infancy and adult skeletal bone health, studies are now assessing single nucleotide polymorphisms with these parameters. When looking at the growth hormone gene (GH1) it was found that homozygotes at specific loci (A5157G and T6331A) displayed lower BMD and had an accelerated rate of bone loss [52]. Interestingly, there was also a statistically significant (p = 0.04) interaction with GH1 genotype, weight at 1 year and rate of bone loss, suggesting susceptibility in the GH/IGF-1 pathway to alterations by environmental influences during intrauterine and early postnatal life.

A further example of a dynamic interaction between genetic and environmental influences of bone mass was shown in the analysis of the vitamin D receptor (VDR) genotype and adult BMD in participants of the Hertfordshire cohort study. There was no significant association between the either birth weight or VDR and adult BMD in the cohort as a whole [53]. However the relationship between lumbar spine BMD and VDR varied according to birth weight. After adjusting for age, gender and weight at baseline it was shown that individuals in the lowest third for birth weight had a higher spine BMD if they were of the BB genotype (p = 0.01). People of the same genotype who were in the highest third for birth weight had a lower spine BMD compared to subjects in the same birth weight category (p = 0.04). This suggests that genetic influences on adult bone size might be modified by nutrition in utero.

In summary maternal behavior, in the form of activities such as smoking, dietary choices including calcium and vitamin D intake, and alterations in the endocrine pathways especially in early life, have been shown to correlate with offspring bone health [48,50,54,55]. The extent to which a particular environment influences the phenotype may depend upon the genotype of that individual [52,53] and the interaction between gene and environment.

### 3.4. Developmental Plasticity in the Natural World

There are striking examples of particular hormones, nutrition and environmental states, during sensitive periods of early life, permanently programming the structure and physiology of non-human animals. The African army worm moth (*Spodoptera exempta*) is an example: as a caterpillar if it grows in an overcrowded, undernourished environment it develops into a moth with a metabolism that mainly uses fatty acids. The fatty acids give energy for long-distance flight, so the moth is better adapted to seek a less crowded environment. If the caterpillar grows in a more hospitable environment then it has a metabolism which is more efficient when staying in the area [56]. In mammals it has been shown that the thickness of the meadow vole’s coat is determined by the amount of light the mother is exposed to prenatally [57]. In this way the vole offspring is better able to adapt for the season it will be born into. The underlying mechanism of the observed variations in phenotype secondary to the experienced early life environment, both in the natural world and in humans, is thought to involve epigenetic mechanisms.

### 3.5. Epigenetics in Animal and Human Studies

Epigenetics refers to changes in phenotype or gene expression caused by mechanisms other than changes in the underlying DNA sequence [58]; each cell in the body acquires a unique “epigenetic signature” reflecting the genotype, developmental history and environmental influences upon the cell [59]. These changes then influence cell function, and so the phenotype of the organism. The two most studied forms of epigenetic marking are DNA methylation and histone modification. DNA methylation involves the addition of a methyl group to cytosine residues at the carbon-5 position of CpG dinucleotides. DNA methylation is generally associated with gene repression, either by decreased binding of transcription factors or by attracting methyl-CpG-binding proteins that act as transcriptional repressors [60,61].

In rats it has been shown that protein restriction during pregnancy is associated with a decrease in the methylation of the glucocorticoid receptor promotor, and a corresponding increase in the expression of the glucocorticoid receptor [62,63]. An effect of this could be an increased sensitivity of osteoblasts to cortisol, which may act to decrease bone mineral density. This would be consistent with the findings of a positive association between birth weight and basal levels of cortisol [49], and the negative association between integrated cortisol concentration and bone density in adults from the Hertfordshire cohort study [50]. Thus the glucocorticoid axis presents an alternative pathway linking maternal nutrition to offspring bone mass.

## 4. Future Work

The role of the early life environment has been shown to have far reaching health consequences in the life of the child, including a significant impact upon their skeletal development. This supports the general need for a programme of interventional research aimed at improving general dietary quality among women before conception and during pregnancy, in addition to studies that evaluate the targeting of specific micronutrients such as vitamin D and calcium.

The Maternal Vitamin D in Osteoporosis Study (MAVIDOS) is a randomized controlled trial to investigate whether supplementation of vitamin D during pregnancy serves to optimize offspring skeletal development, and commenced recruitment in 2008. The study aims to test the hypothesis that vitamin D supplementation of pregnant women who have low levels of vitamin D will result in improved neonatal bone mineral content. This is an ideal platform on which to investigate epigenetic modulation of placental calcium transport, particularly the role of vitamin D. This should improve our understanding of the processes involved in developing osteoporosis, and potentially provide a focus for interventions to avert detrimental processes by influencing maternal dietary habits.

## 5. Conclusion

Osteoporosis is a major cause of morbidity and mortality through its association with fragility fractures. It is becoming increasingly clear that there is a relationship between growth and development in early life and bone health in older age. Sub optimal bone development leads to a reduction in peak bone mass, and a higher risk of osteoporotic fracture in later life. Epigenetic processes may be the mechanism by which the “memory” of early life environment in stored in the offspring. Future understanding of the mechanisms by which maternal diet and behavior influence offspring skeletal development may suggest focused interventions to improve skeletal health throughout the life course, and reduce the burden of osteoporotic fracture in future generations.
